# Spontaneous Cell Cluster Formation in Human iPSC-Derived Neuronal Spheroid Networks Influences Network Activity

**DOI:** 10.1523/ENEURO.0143-22.2022

**Published:** 2022-11-12

**Authors:** Carl-Johan Hörberg, Ulrica Englund Johansson, Fredrik Johansson, David O’Carroll

**Affiliations:** 1 Department of Biology, Lund University, 223 62 Lund, Sweden; 2 Department of Health and Caring Sciences, Linnaeus University, 391 82 Kalmar, Sweden

**Keywords:** 3D neuronal culture, astrocytes, induced pluripotent stem cells, microelectrode arrays, neuronal networks

## Abstract

Three-dimensional neuronal culture systems such as spheroids, organoids, and assembloids constitute a branch of neuronal tissue engineering that has improved our ability to model the human brain in the laboratory. However, the more elaborate the brain model, the more difficult it becomes to study functional properties such as electrical activity at the neuronal level, similar to the challenges of studying neurophysiology *in vivo*. We describe a simple approach to generate self-assembled three-dimensional neuronal spheroid networks with defined human cell composition on microelectrode arrays. Such spheroid networks develop a highly three-dimensional morphology with cell clusters up to 60 μm in thickness and are interconnected by pronounced bundles of neuronal fibers and glial processes. We could reliably record from up to hundreds of neurons simultaneously per culture for ≤90 d. By quantifying the formation of these three-dimensional structures over time, while regularly monitoring electrical activity, we were able to establish a strong link between spheroid morphology and network activity. In particular, the formation of cell clusters accelerates formation and maturation of correlated network activity. Astrocytes both influence electrophysiological network activity as well as accelerate the transition from single cell layers to cluster formation. Higher concentrations of astrocytes also have a strong effect of modulating synchronized network activity. This approach thus represents a practical alternative to often complex and heterogeneous organoids, providing easy access to activity within a brain-like 3D environment.

## Significance Statement

Neuronal “organoid” cultures with multiple cell types grown on elaborate three-dimensional scaffolds have become popular tools to generate brain-like properties *in vitro* but bring with them similar problems concerning access to physiological function as real brain tissue. Here, we developed a new approach to form simple brain-like spheroid networks from human neurons, but using the normal supporting cells of the brain, astrocytes, as the scaffold. By growing these cultures on conventional microelectrode arrays, we were able to observe development of complex patterns of electrical activity for months. Our results highlight how formation of three-dimensional structures accelerated the formation of synchronized neuronal network activity and provide a promising new simple model system for studying interactions between known human cell types *in vitro*.

## Introduction

Recent advancements toward better human brain *in vitro* models hold much promise as the solution to a long history of high failure rates for drug candidates in clinical trials (Paul et al., 2010). Understanding the human brain in health and disease has previously relied heavily on animal-based *in vivo* models and cell culture models, with shortcomings that are partially the reason for the ineffective treatment of many diseases (Paul et al., 2010). This has stimulated development in three-dimensional (3D) *in vitro* model systems that are growing progressively closer to capturing the complex physiological and structural properties of the human brain (Centeno et al., 2018; Zhuang et al., 2018). Among the wide repertoire of 3D neuronal culture systems, organoid cultures are one promising approach with the potential to reduce the need for animal testing and improve prediction of clinical outcomes for novel drugs (Struzyna and Watt, 2021). Organoids are typically derived from a pluripotent stem cell source that is differentiated as cellular aggregates, forming a tissue-like structure where a degree of cellular polarization and compartmentalization is seen (Lancaster, 2013). 3D neuronal culture systems, including organoids, are currently, but not exclusively, used for research into autism spectrum disorders (Russo et al., 2019), Down syndrome (Sobol et al., 2019), schizophrenia (Notaras et al., 2021), and Alzheimer’s disease (Essayan-Perez et al., 2019).

An important consideration when developing a culture model is which cell types are required for the model in question. Many biological systems include complex interaction among multiple cell types, and our ability to model an emergent property of a multicellular tissue thus depends on our representation of the cell populations (Paschos et al., 2015). This perhaps holds especially true when modeling the fast-acting neuronal signaling of the human brain, containing a large number of cell types (Molyneaux et al., 2007) with diverse electrophysiological properties and connective characteristics (Thomson and Lamy, 2007). That being said, it is also true that relatively simple models, which do not take into account the full complexity of the brain, can recapitulate behaviors of mammalian cortex (Traub et al., 1992).

Microelectrode arrays (MEAs) are noninvasive extracellular recording devices that, when integrated into culture substrates, are ideal tools to passively monitor a neuronal culture for very long time periods (Obien et al., 2015). MEAs typically have tens to hundreds of electrodes that measure and amplify electric potentials, enabling the observation of action potentials as well as field potentials over large areas, typically ∼1 mm^2^. MEAs facilitate the long-term tracking of change in neuronal network dynamics, being very useful for the study of toxicological effects of drugs (Tukker et al., 2018) or neuronal development (Trujillo et al., 2019).

While 3D cultures and organoids have already been used to create complex human brain models *in vitro*, they present some challenges akin to that of working with *in vivo* or *ex vivo* brain tissue, particularly for studying electrophysiological interactions with good resolution in space or time. 3D cultures such as hydrogels may require custom-made equipment to facilitate recording (Soscia et al., 2020), or are otherwise dependent on using imaging techniques such as calcium imaging, which suffer from poor temporal resolution (Sevetson et al., 2021). Many heterogeneous organoids pose their own challenges in addition to those of 3D cultures by being highly diverse in their cell compositions (Zafeiriou et al., 2020), thus making them inappropriate for modeling a specific brain region or a smaller subset of cell types.

Here, we present an alternative method for generating self-assembled neuronal 3D cultures, comprised of high-density co-cultures of astrocytes with mature glutamatergic and GABAergic neurons at ratios similar to those in the cerebral cortex. By growing these directly on a standard planar microelectrode array, we could reliably record from up to hundreds of neurons at a given moment for up to 90 d. These developed into spheroid networks with a highly three-dimensional morphology comprised of thick cell clusters, interconnected by pronounced bundles of neuronal fibers and glial processes. We compared two cell densities and two astrocyte densities in a two-by-two experiment design and found that astrocytes are a key driving factor in the self-assembly of spheroid networks. Together with regular microscopic imaging, we were able to demonstrate strong effects of cell cluster formation with network activity and on the spike firing patterns of individual neurons in proximity to the cell clusters. Our approach overcomes some of the challenges of more heterogenous 3D cell cultures and organoids by their defined and controlled cell composition and the reliability of electrophysiological assessment using widely available equipment.

## Materials and Methods

### Chemicals and culture components

Polyethyleneimide 50%, borate buffer, N2 supplement, PBS, 4′,6′-diamidino-2-phenylindole dihydrochloride (DAPI), penicillin-streptomycin, paraformaldehyde (PFA), and laminin were purchased from Sigma-Aldrich. BrainPhys Neuronal Medium was purchased from STEMCELL Technologies. Rabbit anti-GFAP (1:2000; catalog #Z0334, Dako) with secondary antibody (1:200; catalog #AB6800, Abcam); mouse anti-B-III-tubulin (1:200; σT8660) with secondary antibody (1:200; catalog #21202, Thermo Fisher Scientific); rabbit anti-synaptophysin (1:100; catalog #ab32594, Abcam); and mouse anti-PSD96 (1:200; catalog #ab2723, Abcam) used the same secondary antibodies as above. Chicken anti-MAP2 (1:400; catalog #AB5543, Abcam) with secondary (1:200; catalog #A-16039, Thermo Fisher Scientific) and mouse anti-GAD65 (4 μg/ml; catalog #GAD-6-C, Developmental Studies Hybridoma Bank) with secondary (1:200; catalog #21202, Thermo Fisher Scientific) were used.

### Cell cultures

All cell culture substrates (MEAs and glass slides) were coated with polyethylenimine (PEI) 0.07% in borate buffer, followed by 1 h of incubation at 37°C and drying overnight. iCell Astrocytes (ASC-100–020-001-PT) and iCell GlutaNeurons (X1005; FujiFilm Cellular Dynamics International) were thawed according to the supplier instructions, and were counted and tested for viability using trypan blue staining (Table 1). Cultures were subsequently cultured in BrainPhys Neuronal Medium, supplemented with Neural Supplement B and Nervous System Supplement according to the specifications of the suppliers (FUJIFILM Cellular Dynamics, Inc.). The neurons were made up of 83% glutamatergic cells, as determined by quantitative PCR by suppliers, while the remainder were mostly GABAergic cells (supplier, personal communications).

**Table 1:** Seeding densities immediately after seeding and live count taken just before seeding

	High cell density	Low cell density
Total number of neurons	75,000	37,500
Total number of astrocytes	75,000	37,500
Seeding cell density (cells/mm^2^)	5882	2941
Astrocyte live count before seeding	70%	72%
Neuron live count before seeding	62.5	69%

Only live cells were counted in total number of astrocytes and neurons, and thus the seeding cell density and total number of neurons and astrocytes reflects the live cells as included by trypan blue staining.

Cells were seeded onto their respective substrate by application of a 10 μl droplet of cell suspension containing ∼35,000 or 75,000 neurons, depending on the experiment, and a corresponding number of astrocytes to add up to 15% or 50% of astrocytes in total (Table 1). The droplet was incubated for 1 h at 37°C at 95% relative humidity and 5% CO_2_, after which 1 ml of medium was added. Half of the medium volume was changed the following day, after which half of the medium volume was changed three times per week. We performed two sequential seedings, one for low and one for high total cell density. The number of MEAs for the two experiments were 14 for low cell density and 13 for high cell density.

### Electrophysiological recordings

MEAs and associated equipment were acquired from Multi Channel Systems. We used a 60-channel MEA-1060-Inv-BC amplifier with MEAs that had 59 TiN electrodes with 30 μm radius and 200 μm spacing, and referenced recorded potentials against a larger internal reference electrode. Recordings were made at 37°C in room atmosphere. Before recording, cultures were allowed to equilibrate at least 5 min in room atmosphere after being taken out of the incubator.

Spontaneous activity on MEAs (see Fig. 2) was recorded for 10 min at a 32 kHz sampling frequency and 1200× gain. We recorded from all cultures three times per week for the first 48 d, where we changed to recording only twice every week. Data acquisition was performed using MC Rack (Multi Channel Systems), and files were then converted to the HDF5 file format using MC Data Manager (Multi Channel Systems). The exported files were opened in MATLAB (MathWorks) using the third-party publicly available MATLAB plugin MCSMatlabDataTools (2022; Armin Walter, McsMatlabDataTools) where all further analyses were performed using custom-written software.

### Experimental design and statistical analysis

Raw data were filtered using a bandpass (250–3000 Hz) second-order Butterworth filter to filter out slow field potentials, 50 Hz noise, and high-frequency noise. Spikes were detected using an initial double-sided threshold ±5 SDs of estimated background noise. Spikes were sorted into units by principal component analysis (PCA) of detected waveforms. By using k-means clustering, we designed a custom algorithm that finds the likely number of units based on the deviation from the logarithmic decrease in mean cluster radius as a single cluster is divided into increasing numbers of clusters. This is also known as finding the “knee” in scanning k-means. We then applied k-means clustering using the estimated number of units (see Fig. 2).

Basic characteristics, burst detection, network burst (NB) detection, entropy, and correlation analysis were performed using custom algorithms designed in MATLAB. For basic characterization, an active unit was defined as one exhibiting >10 spikes/min, and the mean firing rate (MFR) was calculated from the active units.

Burst detection was performed using the adaptive threshold method, which is described in the study by Pasquale et al. (2010), with minor modifications. To summarize this method, interspike intervals (ISIs) were first used to construct (base 10) log-ISI histograms for every detected unit in each recording. Any existing bimodality was detected according to the method described by Pasquale et al. (2010). The valley separating two peaks of a bimodal ISI distribution was used as a threshold for detecting bursting spikes (i.e., long ISIs were assumed to be associated with interburst periods). Any bursts that contained fewer than three or a total number of spikes divided by 3000 were excluded. If no threshold could be derived from the ISI histogram because of a lack of clear bimodality, it was set to 0.1 s. If the threshold was >0.1 s, only bursts that enclosed a burst detected by a threshold of 0.1 s were accepted.

Network bursts were detected using the temporal overlap of bursts between units on the same MEA. A vector of equal number of samples as the raw recording was created, where every element indicated the number of units that were detected as undergoing a burst at that particular time. A threshold was set as one-third of the mean of the top 10th percentile of this vector. Network bursts that contained less than the total number of bursting spikes divided by 300 or <10 spikes were excluded.

Correlation analysis was performed using the MATLAB built-in cross-correlation functions. Correlation analysis was performed between all units exhibiting >10 spikes/min. The spike trains of each active unit were transformed to log10 inverse ISI vectors, where the inverse ISI was calculated and filled into the entire time span between the two spikes which constitute the ISI. The covariance of units was normalized to their autocorrelation, yielding a correlation coefficient ranging from −1 to 1.

All confidence intervals were derived from bootstrapping (Efron, 1979), where data were resampled with replacement 6000 times, and the means of the resampled sets were used to find the 5th and 95th percentiles, termed from here on confidence intervals.

### Immunocytochemistry

Cells were fixed using 4% PFA in 0.1 m PBS for 2 h, after which they were washed three times with PBS. The cells were permeabilized by incubation overnight in 2% Triton X-100 in PBS (PBST) at room temperature. The cells were then kept in the refrigerator overnight in blocking buffer consisting of 1% Triton X-100 and 10% normal goat serum (catalog #G9023, Sigma-Aldrich). Primary antibody incubation was performed for 2 d in 4°C with antibodies diluted at the fractions indicated in the subsection Chemicals and culture components, in 1% normal goat serum and 0.2% Triton X-100. Next, cells were washed three times with 0.1% Triton X-100 in PBS and left overnight at 4°C. Secondary antibodies in 1% normal goat serum and 0.2% Triton X-100 were applied and left at 4°C for 1 d. Samples were once again washed three times with blocking buffer and left at 4°C overnight. Samples were stained with DAPI (1.5 μg/ml) for an hour and then cleared with RapiClear 1.49 (SUNJin Lab) by incubation at least overnight at 4°C. Samples were mounted on slides and were sealed with nail polish and kept at 4°C until further use. Confocal images were taken using a microscope (model SP8 DLS, Leica) and captured with a 20× oil-immersion objective.

### Image analysis

All phase contrast images were automatically stitched using Fiji. We processed images (see Fig. 7*A*) to remove electrodes from the images by locating black pixels (grayscale value, 0) and pixels with values <1 negative SD from the mean. We applied a smooth mask to those pixels, effectively replacing electrode pixels with the values of adjacent pixels for each color channel (see Fig. 7*B*). Then, we divided the blue values of each pixel by the corresponding red value and applied a bandpass filter to reject smaller features such as individual cells, and any larger features such as variations in illumination. This “hue map” (see Fig. 7*C*) provided us with an accurate indication of where cells had aggregated on the substrate to form larger clusters. To quantify the degree of clustering, we then segmented this hue map by classifying pixels that deviated above the mean by 1.4 SDs as belonging to clusters (see Fig. 7*D*). The segmented image was further processed by erosion to exclude small random pixels. Finally, we calculated the ratio of the number of pixels belonging to identified clusters to the total number of pixels in each given image (i.e., the relative area occupied by large clusters for each image (range, 0–1)], hereafter termed the “cluster ratio” for simplicity.

## Results

### Morphologic development of spheroid networks

**Figure 1.**
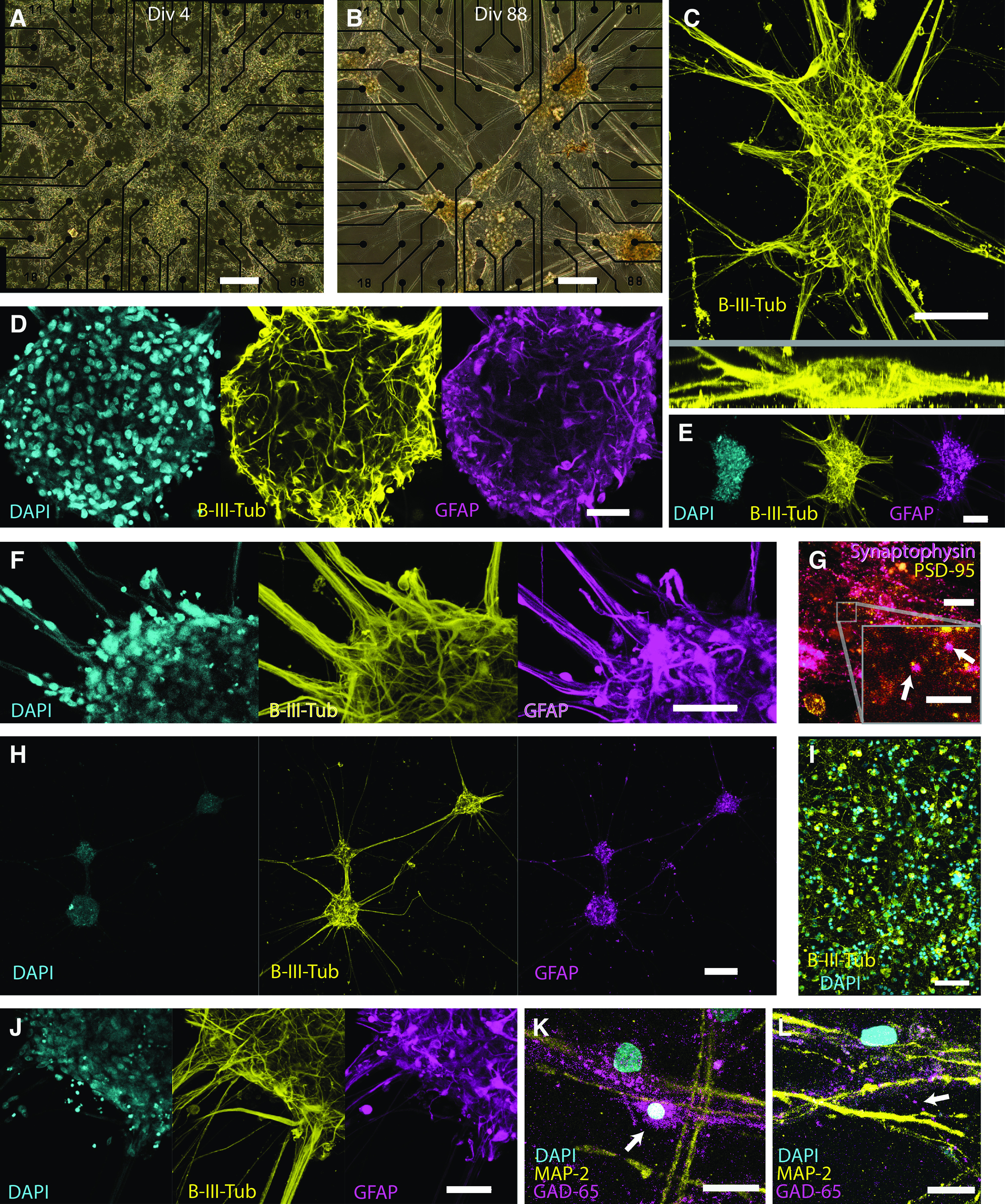
Cell cluster formation and phenotypic characteristics. ***A***, Phase contrast images showing cell distribution and organoids formation. At culture day 4, cells are spread out across the electrode area. ***B***, At 88 d, distinct clusters are formed, with distinct processes interdigitating the clusters. ***C***, Confocal maximum intensity projections (MIPs) of fluorescent immunolabeled B-III-tubulin, a marker for neurons: top (top) and side (bottom) views of a representative cluster. Cluster width often exceeds 100 μm and thickness can be up to 60 μm. ***D***, Confocal cross section of a large cluster, showing that fluorescently labeled neuronal (B-III-tubulin, yellow), astrocyte (GFAP, magenta), and nucleic (DAPI, cyan) markers are found throughout the volume of the clusters. ***E***, Confocal MIPs of fluorescently labeled neurons (B-III-tubulin, yellow), astrocytes (GFAP, magenta), and nuclei (DAPI, cyan). ***F***, Confocal MIP of fluorescently stained neurons (B-III-tubulin, yellow), astrocytes (GFAP, magenta), and nuclei (DAPI, cyan) at the base of the projections that interdigitate the clusters. Neurons and glia are typically seen growing in very tight association both in projections and in clusters. ***G***, DIV 34 fluorescently stained postsynaptic and presynaptic markers PSD-95 (yellow) and synaptophysin (magenta) respectively show rich expression of both markers. Inset, The two markers are occasionally observed a small distance apart from each other, showing that putative NMDA receptors are forming at synapses. ***H***, Confocal MIPs of fluorescently stained neurons (B-III-tubulin, yellow), astrocytes (GFAP, magenta), and nuclei (DAPI, cyan), showing how clusters organize with interdigitating projections. ***I***, Confocal MIPs of fluorescently stained neurons growing in the absence of astrocytes at 14 DIV, showing no clustering. ***J***, Confocal MIPs of fluorescently stained neurons (B-III-tubulin, yellow), astrocytes (GFAP, magenta), and nuclei (DAPI, cyan), showing the base of the projections. It is clear that the projections are sparsely populated by nuclei, which mostly reside inside clusters. ***K***, Confocal MIPs of fluorescently stained DAPI (cyan), MAP-2 (yellow), and GAD-65 (magenta) showing two neurons; one indicated by an arrow is expressing GAD-65, which was classified as a GABAergic neuron. ***L***, Confocal MIPs of fluorescently stained DAPI (cyan), MAP-2 (yellow), and GAD-65 (magenta) indicated by an arrow showing a band of GAD-65 puncta, possibly from a GABAergic synapses. Scale bars: ***A***, 200 μm; ***B***, 200 μm; ***C***, 100 μm; ***D***, 50 μm; ***E***, 100 μm; ***F***, 50 μm; ***G***; 20 μm; inset. 2.3 μm; ***H***, 200 μm; ***I***, 150 μm; ***J***, 50 μm; ***K***, 20 μm; ***L***, μm.

In the first days after seeding, cells appear as a mostly uniform single-cell layer, as seen in phase contrast microscopy (Fig. 1*A*), taking up an estimated average area of 25.5 mm^2^, resulting in initial seeding densities as stated in the Table 1. Over a period of weeks, however, the cells migrate to form dense clusters, ranging up to 200 μm in diameter (Fig. 1*B*). Cluster formation often progress from several smaller and less pronounced clusters, which then fuse gradually to create larger clusters. Typically, clusters coincide with considerable clearing of adjacent areas, leaving behind thin nerve-like strands that interconnect the clusters, forming what we call “spheroid networks.” Individual clusters remain highly mobile throughout culture, and often move in and out from the effective recording range of electrodes. The rate of cluster formation was not identical between and within the different culture conditions, an observation that we discuss in more detail below (see subsection Astrocyte density and spheroid network formation).

To investigate whether the interior of the clusters contained a necrotic core or cellular compartmentalization, we used confocal imaging and tissue clearing to create optical sections of immunolabeled clusters (Fig. 1*C–J*). Staining for the neuronal marker B-III-tubulin and the astrocyte marker GFAP, and DAPI staining of nuclei were seen throughout these structures, thus revealing no evidence of a necrotic core inside the clusters. On the contrary, optical sections show dense neuronal networks, glial projections, and nuclei throughout the spheroid network (Fig. 1*D*). We could also not find evidence of any clear compartmentalization or vertical stratification of neurons or astrocytes in any sample. Quite the opposite, we found that astrocytes and neurons are highly integrated, showing an almost identical distribution, differing only in individual cell projections and somas (Fig. 1*F*). We frequently observed clusters with a thickness >60 μm (Fig. 1*C*). We estimated cell density in a small volume inside three clusters by manual counting of DAPI-stained nuclei (Fig. 1*D*). Cell density was on average 447,000 cells/mm^3^, higher but within the same order of magnitude as estimates of cell density of human cortex (Cotter et al., 2001; Rajkowska et al., 2001). Direct comparisons are, however, difficult because of the heterogenicity in the cell distribution in cortical tissue.

The cells often organize themselves into discrete clusters, leaving behind bundles of neuronal and glial projections (Fig. 1*H*), which interconnect the clusters. These projections are sparse in cell nuclei, mostly consisting of neuronal and glial projections (Fig. 1*J*), and so are analogous to neuronal tracts that interconnect spatially unique brain regions or neuronal ensembles that communicate through long axonal projections. We never observed separate clusters exhibiting dissociated or uncorrelated network activity (see subsection Synchronized network bursts), but we could show that this morphology has a strong effect on network activity (see subsection Spheroid network formation influences burst development).

To examine the formation of synapses, we used additional immunolabelling of synaptophysin, a synaptic vesicle protein found in synaptic preterminals (Tarsa and Goda, 2002), and PSD-95, a label for postsynaptic densities associated with NMDA receptor regulation (Won et al., 2016). We observed an abundance of both proteins (Fig. 1*G*), with occasional colocalization of both presynaptic and postsynaptic markers (Fig. 1*G*, inset) when performing immunofluorescent staining at 34 d *in vitro*.

To validate the presence and quantity of GABAergic neurons, we stained for MAP-2, a general neuronal marker, and GAD-65, which is specific for GABAergic neurons. This way, we could validate the presence of GABAergic neurons by the presence of double-expressing cells (Fig. 1*K*), but also an abundance of projections and puncta, indicative of GABAergic synapses and axons (Fig. 1*L*). We classified neurons based on the criteria of expressing MAP-2, having a single nuclei, and by a clear association with a projection. This excluded many cells that were either too densely packed or too faint to meet all criteria, but 36 neurons could be classified with confidence. In turn, a subset of GABAergic neurons was classified through the additional expression of GAD-65, throughout the soma but also with some degree of visible projection, as GAD-65 is expressed in synapses of GABAergic neurons. This way, we could detect seven GABAergic neurons giving a proportion of 19% GABAergic neurons, consistent with the analysis of the supplier that the neurons were an 83% glutamatergic population, with GABAergic neurons forming the remainder (FUJIFILM Cellular Dynamics, Inc., personal communications; see subsection Cell cultures). Despite the small number of neurons counted, it is clear that GABAergic neurons are present and forming what appear to be synapses.

### Development of burst firing

**Figure 2.**
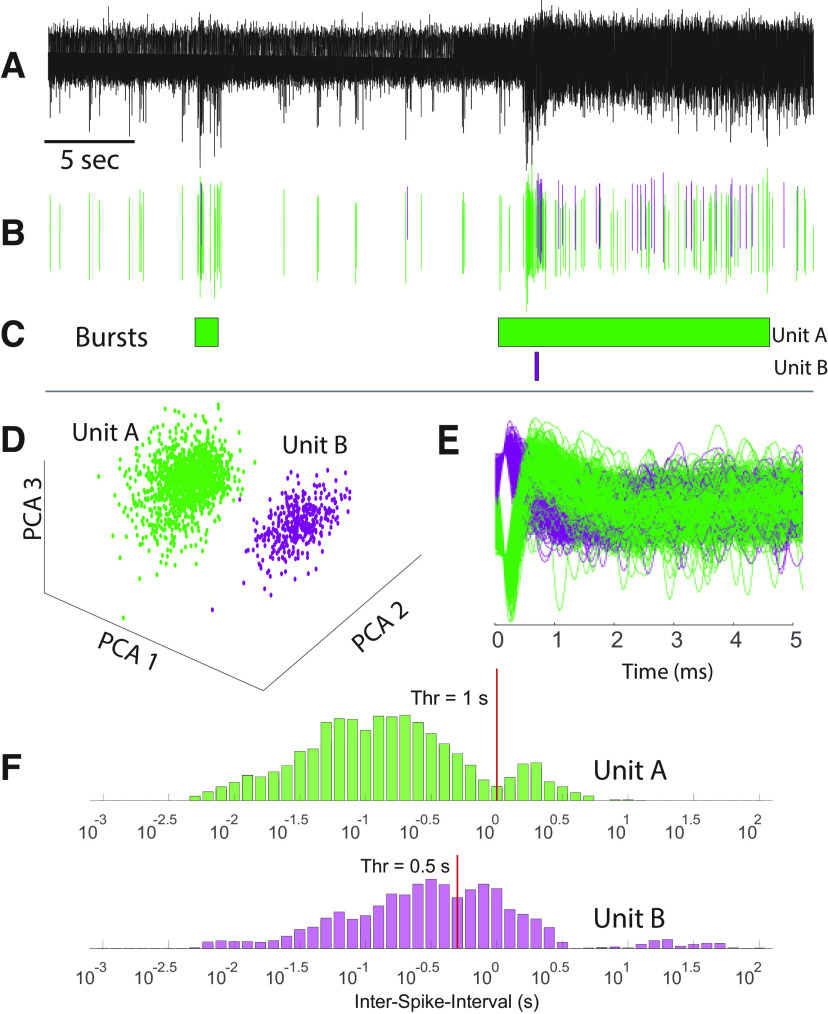
Principle for spike sorting and burst detection. ***A***, Example of filtered data from one electrode in a 25-d-old culture. Spontaneous activity in the form of spikes is seen throughout the recording. Two bursts of variable durations are visible in this segment. ***B***, Sorted spike waveforms for two different units are colored by the unit of origin. ***C***, Colored boxes showing periods identified as bursts for the two different units seen in ***A*** and ***B***. ***D***, PCA of spikes obtained from a filtered signal. Units appear as distinct clusters in PCA space, and are potentially spikes from two different neurons. ***E***, Aligned spike waveforms showing waveforms of classified units, highlighting the difference in the shape of action potentials for the two units. ***F***, Determination of threshold for burst detection. A separation between two modes of a log ISI histogram is identified (vertical lines) and used as a threshold for burst detection.

**Figure 3.**
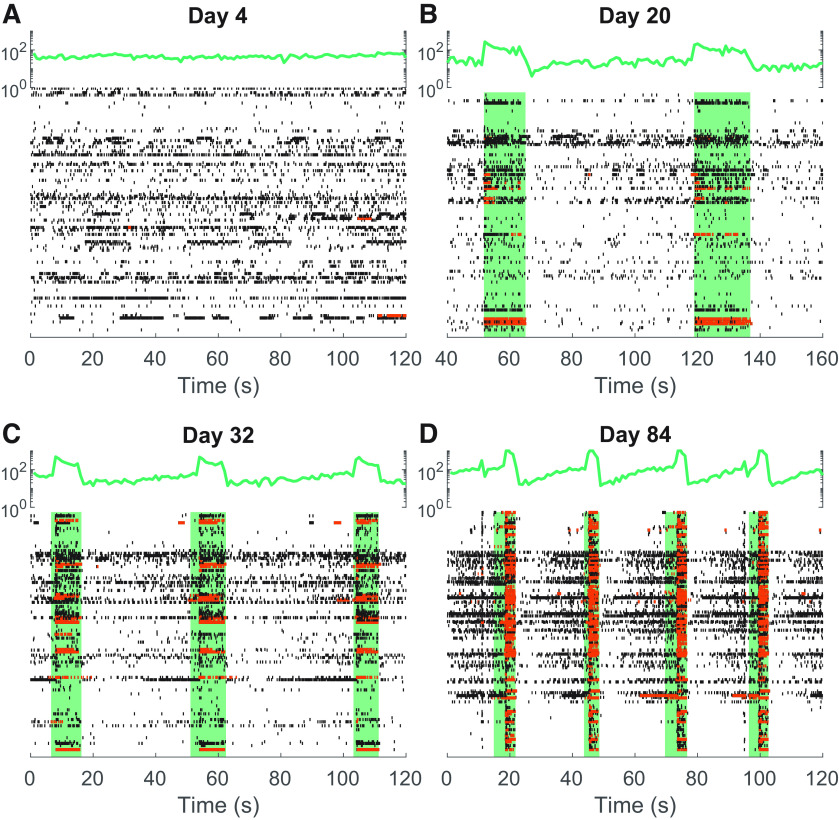
Raster plots from an example MEA recording showing detected spikes for all electrodes individually (black and red dots) and summed spikes across all electrodes binned into 100 ms bins (green line). Spikes classified within bursts are highlighted in red. When bursts overlap sufficiently between units, they are classified as network bursts, shown here as green boxes. ***A***, Day 4 recording showing spontaneous asynchronous activity on many electrodes. ***B***, At ∼3 weeks, characteristic busts are seen, which results in an increased spike rate on electrodes synchronously, giving rise to a peak in summed binned spike rate. ***C***, ***D***, As cultures continue to mature, the dynamics of network bursts change, typically increasing in occurrence frequency and decreasing in duration.

Some basic features of the development of electrical activity were consistent across MEAs at ratios of both the astrocyte and neuron densities tested. Spontaneous spikes were already seen on many electrodes at 4 d after seeding [i.e., days *in vitro* (DIV)], although note that in this case the term “days *in vitro*” is relative to the seeding date since these commercial cell lines were already predifferentiated into specific cell type linages). This early activity is characterized by low spontaneous firing rates, with spikes that occur independently on each electrode but often at very regular intervals, typically a few spikes per second. As the cultures progress and mature, the spike patterns become less regular while also showing higher spike rates (Figs. 2, 3). Within 1–2 weeks after seeding, the pattern changes to one where spikes tend to occur in bursts—periods where the firing rate is sustained at, for example, >10 spikes/s and of several seconds duration, interspersed by longer periods of lower spike rate (“interburst intervals”), often lasting up to 10 s. This bursting becomes more pronounced over time and eventually transitions to a state where bursts occur synchronously on all active channels of a given MEA, a feature we define as “network bursts (NB).”

To quantify and characterize these changes in network activity, we first detected individual event waveforms on each electrode at a threshold of ±5 SDs of estimated background noise levels, set individually for every electrode (Fig. 2*A*,*B*). Spikes were sorted into units using PCA and a custom k-means-based unsupervised clustering approach implemented in MATLAB (Fig. 2*D*,*E*) in an attempt to discriminate spike trains from multiple neurons on each electrode. Following unit assignment, we then determined the occurrence of bursts for each unit using an adaptive threshold based on the log interspike interval histogram (Pasquale et al., 2010; see Materials and Methods). Interspike intervals derived from all spikes for each unit allowed construction of a histogram (LogISI histogram; Fig. 2*F*) representing the probability distribution of the intervals. Bursting behavior is expected to yield bimodal distributions in LogISI histograms, whereby the lower mode (shorter intervals) is likely to represent spikes that occur during a burst, while the larger mode represents spikes mainly from the interburst period, as is clearly the case for Unit A (green) in Figure 2*F*. The valley separating the two modes (Fig. 2*F*, red line) is thus an appropriate threshold to segregate these two spike subpopulations. We detected this valley using an unsupervised algorithm, as described in the study by Pasquale et al. (2010), and then considered any spikes with an ISI below this threshold for potential inclusion within a burst, allowing us to then parse individual spike trains to define the start and end of each burst (see Materials and Methods).

**Figure 4.**
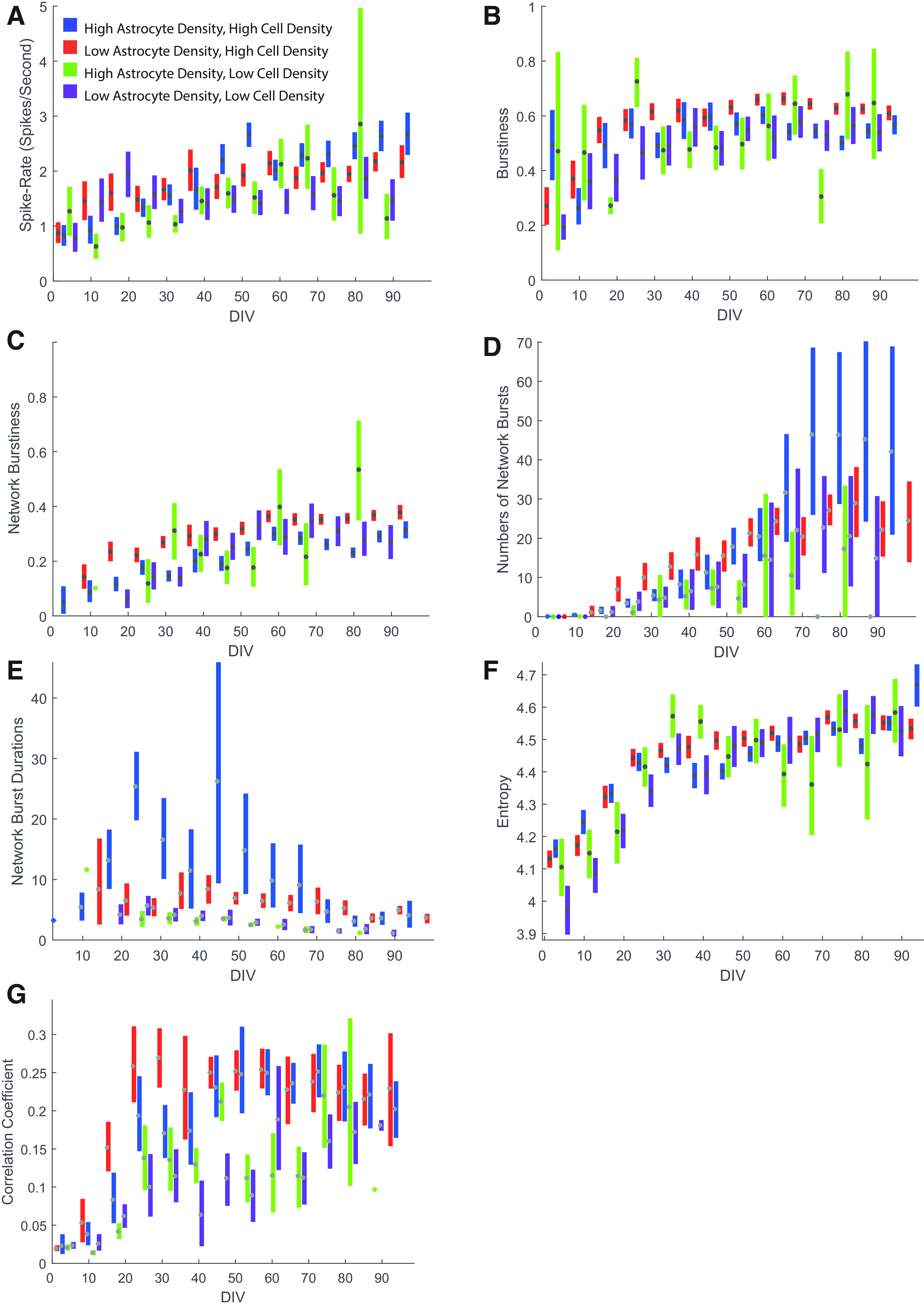
Network characteristics of neuronal cultures. All figures show confidence intervals obtained from bootstrapping. ***A***, Overall activity in spikes per second for active units and four different culture compositions. ***B***, A burstiness metric (see Materials and Methods) giving the ratio of spikes occurring during bursts to total number of spikes. ***C***, Network burstiness metric giving the fraction of total spikes occurring during network burst events. ***D***, Number of network bursts per 10 min recording. ***E***, Duration of network bursts. ***F***, Entropy of spike trains. ***G***, Correlation coefficient in spike rates between units on the same MEA.

Figure 3 shows raster plots for short windows of the recorded activity of individual units from an example MEA at four different time points over the 3 months of culture. Very few burst periods meeting our criteria were identified at day 4 (Fig. 3*A*, orange dots), while by day 84, the majority of units are seen to generate distinct bursts (Fig. 3*D*). The relative prevalence of these bursts was determined by dividing the number of bursting spikes within these periods by the total number of spikes, a metric that we refer to as “burstiness” from here on, such that a value of 1.0 would represent a unit where spikes only occurred in bursts. In Figure 4, we have quantified how this and other metrics change over time across multiple MEAs for all four culture conditions, using a bootstrapping method to define the distributions and confidence intervals of values for each time point. All four culture compositions show a gradual increase in both MFR and burstiness (Fig. 4*A*,*B*) with an apparent plateau to steady state after 30 d. Although all four culture conditions showed this same basic pattern, bursts were in general more prevalent in low-astrocyte ratio cultures with high density, and less so in cultures with low cell density.

### Synchronized network bursts

As cultures mature, bursts begin to occur strikingly synchronized on nearly all active channels simultaneously (Fig. 3). To quantify the onset and duration of these NBs, we expanded on the burst detection algorithm described above to identify co-occurring bursts on multiple channels (i.e., synchronized bursts). We first defined periods (Fig. 3, green bands) where most units on the MEA enter a bursting phase, using an algorithm described in more detail in Materials and Methods. Similar to our individual unit burstiness metric, we then also defined the relative prevalence of NBs as the number of NB spikes divided by the total number of spikes for every unit, giving us a “network burstiness” metric.

Compared with individual neuron burstiness, network burstiness does not appear as early in culture, with both the network burstiness metric (Fig. 4*C*) and the total number of network bursts (Fig. 4*D*) increasing over time throughout culture over all four conditions, with a less evident plateau after 30 d. As with burstiness, high-density cultures generally showed greater network burstiness compared with low cell density, with low astrocyte ratio at high cell density showing the highest network burstiness. Corresponding to this general increase in network bursting, the average duration of network bursts (Fig. 4*E*) tends to decline over time, with the longest durations seen initially in the high-astrocyte, high-cell density condition, but declining over all four culture conditions to <5 s after 70 d in culture.

### Entropy

As noted earlier, during the first few days of culture, before network bursting appears, we often observed individual neurons with near-constant firing rates (i.e., with a single narrow mode in the ISI histograms). Once the network develops to exhibit network bursts, however, the ISI histograms typically become more complex, indicative of the more structured spike-firing patterns associated with burst and interburst periods described above. The apparent complexity of spike patterns continued to increase at later time points, however, with highly variable patterns of spikes also occurring in the periods between network burst events. As this feature would not be captured by our burst or network burst metrics, we further asked whether entropy could be a complementary metric to assess network behavior. We calculated this using Shannon’s approach to estimate entropy as follows:

(1) $$\text{Entropy}=-\overset{N}{\underset{i=1}{\sum }}\log\left(p_{i}\right)*p_{i},$$

where *p*_i_ is the binned discrete probability density function of the logISI histogram, normalized as follows:

(2) $$\overset{N}{\underset{i=1}{\sum }}p_{i}=1.$$

This way, entropy captures the randomness in spike firing: a neuron exhibiting only one ISI (i.e., if all spikes occurred like the ticks of a clock at a steady and constant rate) would yield the lowest possible entropy value, zero, while neurons where the ISI is highly variable would show a higher entropy. This is fundamentally different from a simpler metric such as the SD of ISI; however, since it distinguishes neurons that might have two simple modes (e.g., simple bursting, because of turning on or off a simple excitatory synaptic input) from one where responses are less clearly stereotyped, as might be expected when there are more complex synaptic interactions. In the former case, all spikes would be at either a very low or very large ISI, so entropy would remain low despite the large spread in ISIs. Figure 4*F* shows this metric computed across all culture conditions and day points. Entropy increases in a pattern similar to that of burstiness, increasing rapidly up to ∼30 d, where it begins to taper off. Like burstiness, this metric is computed on a per-unit basis. Unlike burstiness, however, entropy continues to increase for as long as the experiment continued, reflecting a continuous increase in the firing complexity not evident from simple burst analysis. Before day 30, this metric reveals a clear distinction between high-density and low-density cultures, which show high and low entropy, respectively. This distinction is mostly gone by day 30, where cultures appear to reach a similar but dynamic degree of entropy, unlike burstiness where low astrocyte ratios in high-density cultures were more consistently bursty throughout the course of culture. This shows that entropy analysis can be a useful tool to detect features of network activity that would otherwise not be detected by typical burst detection algorithms.

### Correlation analysis

**Figure 5.**
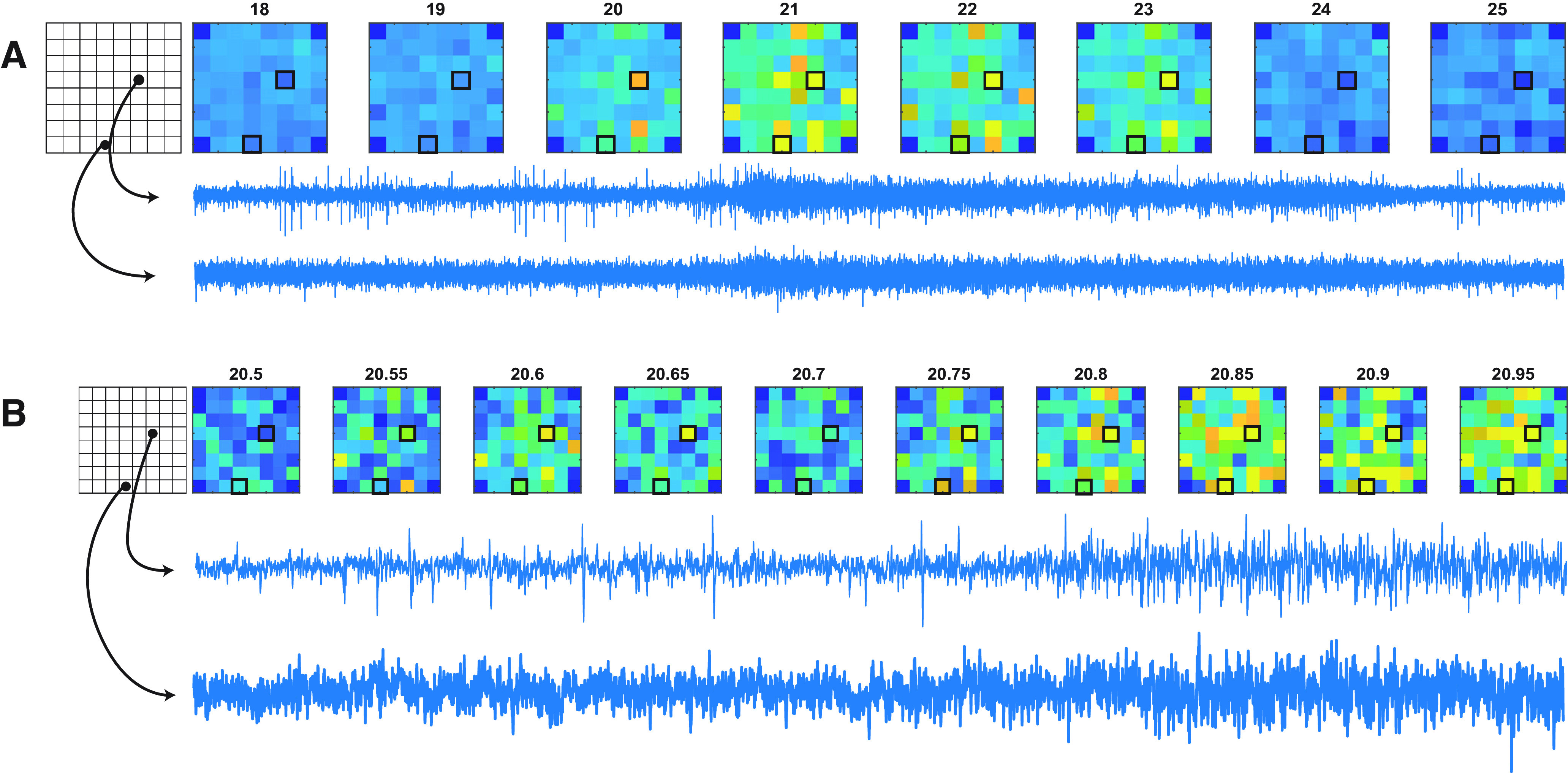
Activity maps for all channels of a MEA during a network burst. Colors show the amplitude envelope of the filtered signal averaged across the time points indicated above each pictogram. ***A***, The network burst typically includes a large subset of electrodes and often shows some response on every electrode. ***B***, The same network burst as in ***A***, but at a finer temporal scale. This highlights the characteristic “fuzziness” of an electrode during a network burst event. Network burst events typically occur simultaneously on almost all channels, with little to no obvious initiation site.

The increase in network burstiness over time that we observed is suggestive of an ongoing maturation of synchronous activity mediated by extensive synaptic coupling between neurons to generate a network. As cultures developed beyond 2–3 weeks, we observed correlated spike rates between channels across entire MEAs (i.e., where, at least at burst onset, most neurons recorded were simultaneously bursting; Fig. 3*D*). This phenomenon is illustrated by Figure 5, which shows “heat maps” for MEA neuronal activity before, during, and after a network burst (Fig. 5*A*). In this example, there is no obvious focus for the onset of activity that then spreads elsewhere (Fig. 5*B*). Rather, individual electrodes at distant locations on the MEA become simultaneously active within a few tens of milliseconds. This is consistent with possible long-range synaptic coupling mediated via the “nerve-like” bundles of parallel fibers described in Figure 1, which may span clusters located on electrodes that are hundreds of micrometers apart.

**Figure 6.**
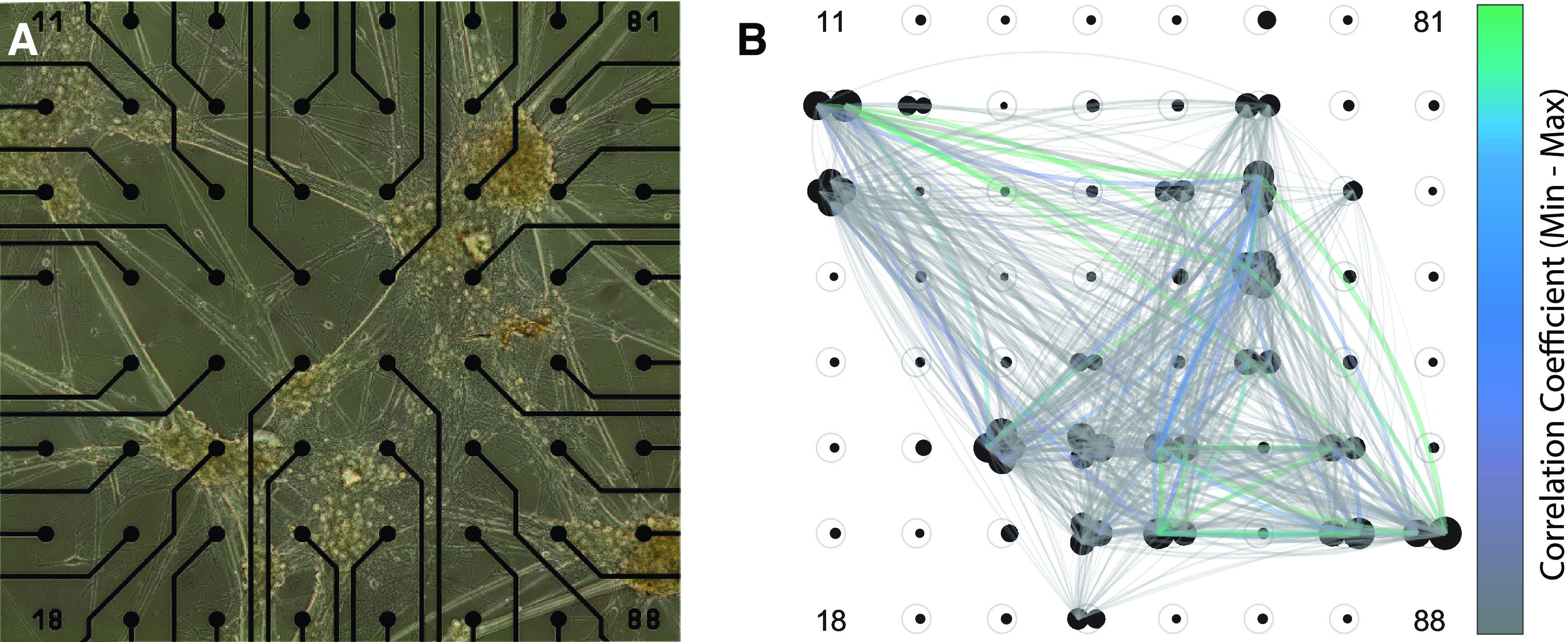
An example showing correlation between units on each electrode. ***A***, A phase contrast image montage taken immediately after the recording. ***B***, Black circles represent individual neurons sorted from the electrodes, with diameter scaled to the relative activity (in spikes per second). The curved line width and color are proportional to the correlation coefficient between each unit pair. We see that strong correlations are not confined to local clusters of cells, but span long distances, which morphologically appear only to be connected by the characteristic “strands” or “bundles” of glial and axonal projections.

Measuring neuronal correlation has often been interpreted as an indicator of synaptic connectivity (Chiappalone et al., 2006; Brewer et al., 2009). We quantified this correlation between neurons by first transforming spike trains for each unit to instantaneous spike rate (inverse ISI) vectors sampled onto a discrete time base where all samples between consecutive spikes were set to the inverse of the interspike interval. This was then downsampled to a sample rate of 320 Hz. The logarithm of this vector was then used to calculate the correlation coefficient between unit pairs on all electrodes. We excluded unit pairs from the same electrodes to ignore spurious negative correlations resulting from spike-sorting artifacts. Figure 4*G* shows the distributions for this correlation across the four culture conditions and all time points. The first 2 weeks were dominated by weakly correlated or uncorrelated activity in all culture types, where neurons fire mostly independent of each other. After 3 weeks, at the time when pronounced network bursts are first seen, this is reflected by a rapid increase in average correlation coefficients. Note that strong correlation is not only dependent on bursts, but may also reflect correlated changes such as the silent periods after network bursts (Fig. 3*D*). With this is mind, it is interesting to note a clear discrepancy between high-density and low-density cultures, with high cell densities showing both earlier emergence of strong correlation and sustained greater correlation even after several months. This difference, less evident in our other quantified network metrics, suggests an increased ability to form a strongly connected network in high-cell density cultures, and in particular when astrocyte ratios are lower. Importantly, correlated activity is not confined to local circuits, but span the entire active recording area, as seen in Figure 5. We illustrate this for one MEA in Figure 6, where we show the clusters on the MEA (Fig. 6*A*) together with the correlation between pairs of active units as colored lines linking electrodes (Fig. 6*B*). This shows no obvious relationship between the strength of correlation and proximity between active channels (with mean firing rate shown as the diameter of the black markers for each unit at each location), with many of the best correlated electrode pairs having among the largest physical separations on the active area of the MEA.

### Astrocyte density and spheroid network formation

**Figure 7.**
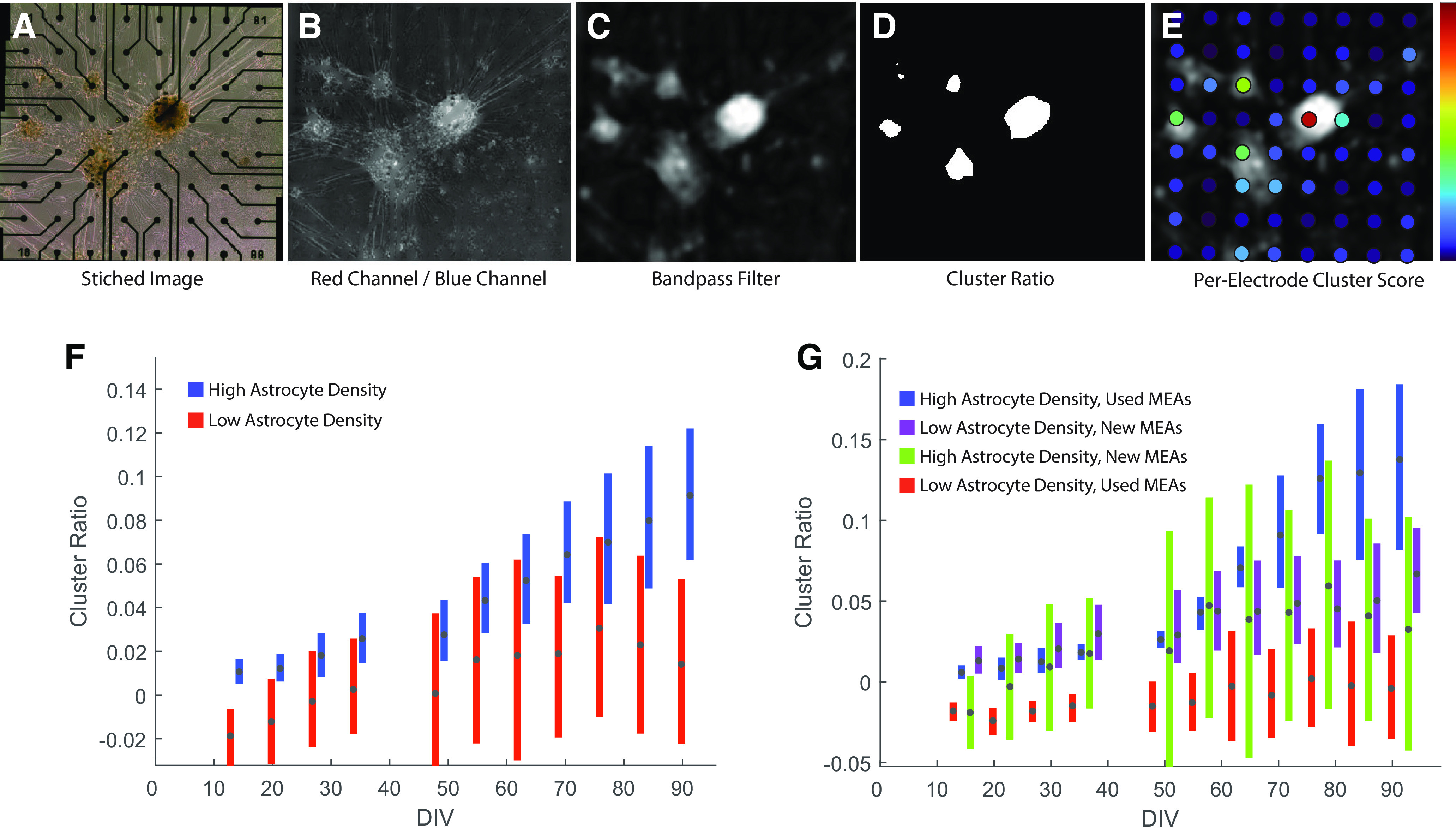
Quantifying cluster formation. ***A***, Stitched montages from phase contrast images show that organoids have a brownish tint. ***B***, Dividing the red value of every pixel with the corresponding blue value gives a new image that indicates relative values of red and blue. Electrodes were removed simply by finding very dark pixels and effectively applying pixel values of nearby pixels to electrode pixels. ***C***, The background light intensity sometimes varies across the full stitched image, so a bandpass filter was applied, which also removes uninteresting small features. This bandpass-filtered image was used in two approaches. ***D***, ***E***, Simple segmentation to acquire the degree of cluster formation over the entire electrode array (***D***), and estimating a per-electrode clusterness metric or cluster score of the proximity of every electrode individually (***E***). The latter was obtained by multiplying a small Gaussian kernel on the positions of the electrodes. ***F***, Cluster ratio normalized to the cluster ratio of the first day. When comparing high astrocyte and low astrocyte ratios, we can see that high astrocyte ratios tend to cluster more and faster. ***G***, Cluster ratio normalized to the cluster ratio of the first day, showing that used MEAs develops faster in high astrocyte ratios than in low astrocyte ratios. The difference is not seen on new MEAs, which show greater cluster formation than the low astrocyte ratio in used MEAs.

We observed that cultures with higher astrocyte ratios seemed to develop clusters faster than those with low astrocyte ratios, but also that newer MEAs promoted cluster formation, and we wanted to quantify this observation. To quantify the degree at which cells formed clusters, we processed images (see Materials and Methods) to take advantage of a red shift in hue caused by light absorption when cells migrate into dense clusters and to map areas occupied by such clusters (Fig. 7*A–E*). This allowed us to compute a “cluster ratio” (i.e., an estimate of the relative area of the MEA occupied by large clusters). We then calculated confidence intervals, as described previously using the bootstrap method, for each time point and for each culture condition (Fig. 7*F*,*G*). This way, it is evident that astrocyte concentration has a strong impact on cluster formation (Fig. 7*F*). For this analysis, we further subdivided the data to quantify the effect of the prior culture history for individual MEAs on the rate of cluster formation. Fresh MEAs are initially hydrophobic, and the MEA manufacturer recommends a number of potential treatments to promote cell adhesion, including growing and discarding an initial cell culture, as well as the surface treatments such as those that we applied (coating with PEI and laminin). Even so, during long-term culture, it is likely that surface properties continue to evolve because of prolonged interaction with cultured cells and medium. On used MEAs, the astrocyte density had a strong positive impact on cluster formation (Fig. 7*F*), where cluster ratio has been normalized for each MEA, based on their initial cluster value.

### Spheroid network formation influences burst development

During the course of our experiment, we observed that electrodes that recorded directly from large clusters showed more pronounced bursting behavior, while electrodes from more peripheral locations typically show a more regular pattern of spiking even in mature cultures, reminiscent of that seen at early day points. This suggests that bursting is correlated with the development of spheroid networks, presumably because of the emergence of synaptic coupling that develops mainly within clusters. We tested this hypothesis by using the images that we segmented to identify electrode areas with large clusters (Fig. 7) to also estimate the degree of cluster formation in the immediate proximity of every individual electrode. After identifying the position of every electrode from the original phase contrast images, we multiplied the hue map for the pixels in the vicinity (31 × 31 μm) of every electrode with a Gaussian mask (1 SD) to obtain a per-electrode “clusterness” metric (i.e., an electrode-centered weighting for the degree of nearby large cluster formation at each electrode; Fig. 7*E*).

**Figure 8.**
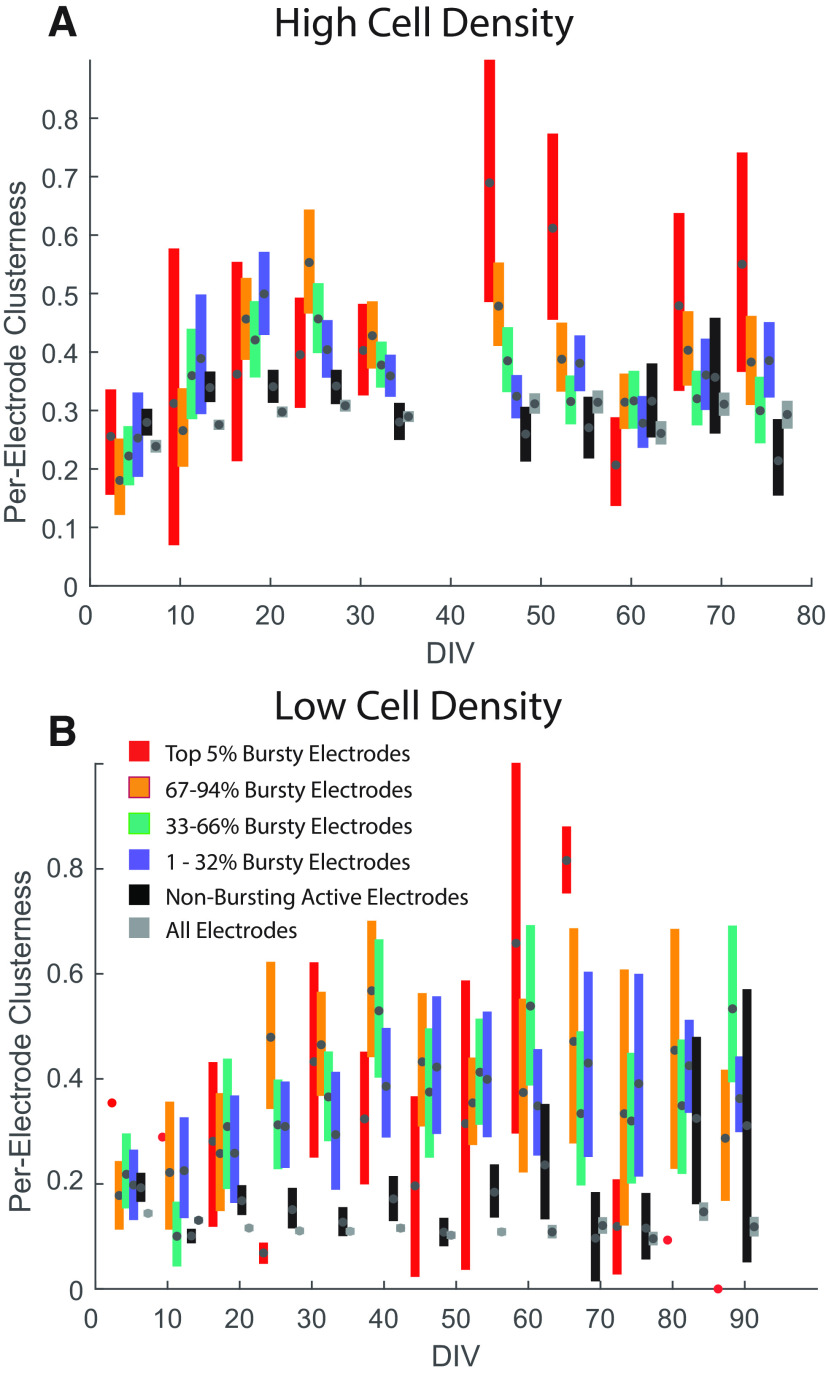
Relationship between cluster formation and burstiness for all electrodes of all MEAs of high and low cell density. ***A***, At high cell density, we often see that the per-electrode clusterness is different depending on how bursty the electrodes are. This effect appears after the third week and is sustained through most of the full duration of the culture cycle. In general, the most bursty electrodes from all MEAs show higher cluster scores, meaning that the bursty electrodes are commonly found where cell clusters are the largest and most pronounced. ***B***, This effect is only occasionally seen in low cell density, likely because of the smaller sample size of this dataset, as low cell density often shows a smaller number of active electrodes.

We plotted pooled data (i.e., independent of age or astrocyte density) for our per-electrode clusterness metric against time (Fig. 8). We divided our data into four groups based on our earlier analysis of their degree of burstiness (Fig. 4*B*) from the most bursty (top 5% burstiness) to least bursty (1st to 3rd percentile of burstiness), plus an additional group for electrodes that were completely “nonbursting” (i.e., where no per-unit bursts were identified). For high cell density (Fig. 8*A*), these data show a clear trend toward more bursty electrodes being associated with higher cluster scores, with the 95% confidence intervals for the top 5% most bursty electrodes being substantially higher than for less bursty electrodes. This confirms our observation that electrodes that record directly from a large cluster typically exhibit more pronounced bursting behavior. The relationship is particularly prominent after 1 month of culture, and generally less so in early and late stages of culture. In lower-cell density cultures, this effect is less obvious (Fig. 8*B*), although again the nonbursting electrodes at least were rarely associated with clusters. The weaker effect here can at least in part be explained by the smaller sample size of low-cell density cultures.

### Relationship between cluster development and correlated network activity

**Figure 9.**
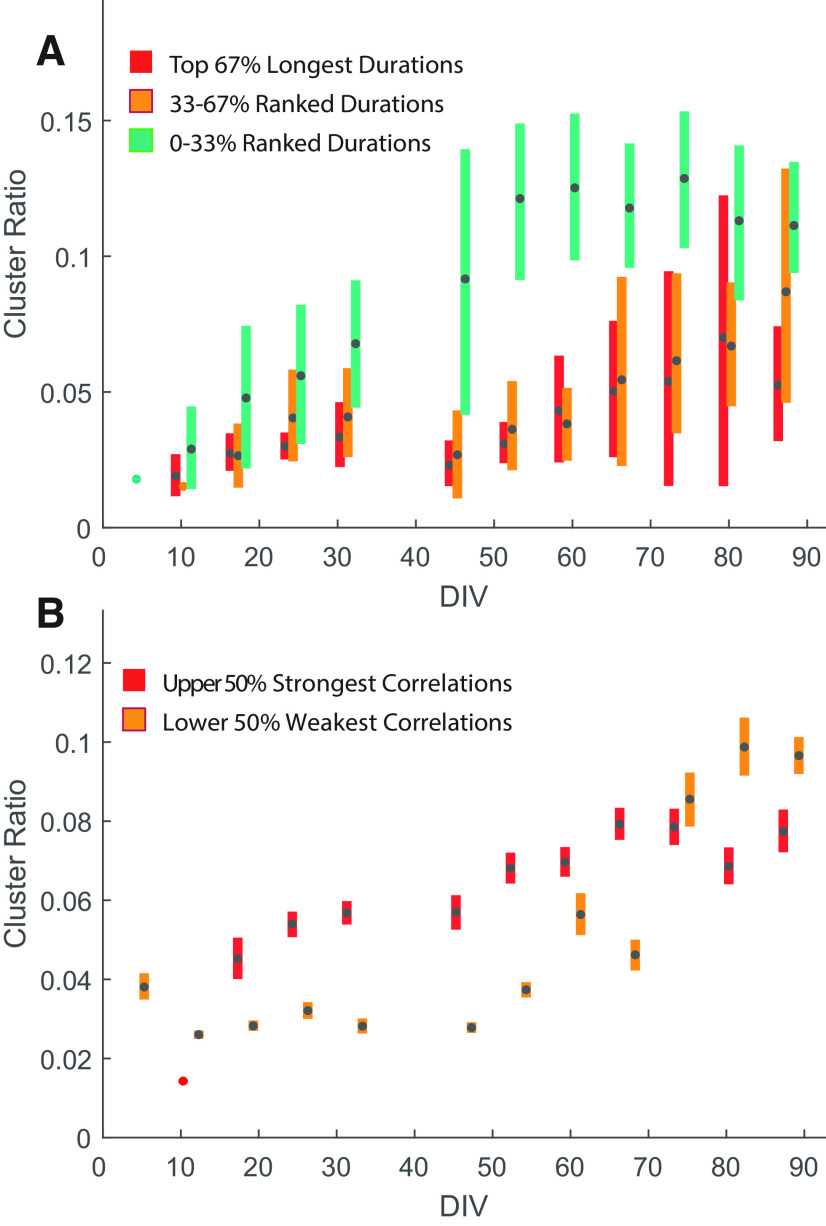
Effect of cluster formation on network burst durations and correlation coefficient. The cluster ratio was derived from the ratio between pixels segmented as being part of a cluster to the total number of pixels and represents the extent that clusters have formed on individual MEAs. ***A***, Network bursts durations, averaged across one recording for all MEAs, are influenced by the extent to which the MEAs have formed clusters. The lower tertile (0−33%) of burst durations are relatively more confined to MEAs, which have formed pronounced cluster (i.e., cluster formation negatively influences network burst durations). ***B***, Correlation coefficient, again averaged for all unit pairs on individual MEAs, is also influenced by cluster formation. Correlation coefficients above the median of all MEAs are generally confined to MEAs with stronger cluster score. This implies that cluster formation is positively correlated with the formation of strongly correlated networks of neurons.

Our data are consistent with the hypothesis that neurons that reside in highly populated areas receive more synaptic inputs than neurons that reside in peripheral areas, leading to local feedback circuits capable of generating local bursts of spikes. These could in turn entrain network bursts via the nerve-like interconnections between clusters. Similar observations have been reported when experimenters have compared 3D to 2D neuronal cultures, where 3D cultures showed clearer network bursts with long durations (Frega et al., 2014). In our pilot experiments, we observed that cultures that rapidly form clusters (particularly on fresh MEAs) also tend to show accelerated development of correlated network burst activity. We wondered whether this effect could be quantified by our analyses of clustering and the correlation between electrodes. We thus took the MEA-wide cluster ratio, derived from segmented images (Fig. 7*D*), to estimate the degree of cluster formation for every individual MEA over time. We then divided all MEAs from a given recording date into tertiles based on the mean network burst duration (Fig. 9*A*). From these data, we can clearly see that the MEAs with the shortest network bursts were significantly more clustered, while longer network bursts were largely confined to MEAs with less cluster formation. We further divided MEAs into two quantiles based only on the mean correlation coefficients between unit pairs and plotted them against cluster ratio (Fig. 9*B*). We see a strong effect of cluster formation on correlated activity, with the upper higher correlations between electrodes showing a greater degree of cluster formation (Fig. 9*B*), particularly in the early days after culture, but persisting for at least the first 2 months of culture. By the third month, clustering is so ubiquitous (independent of the culture conditions) that most MEAs showed strong average correlation between electrodes. This supports the notion that strongly correlated network bursting is associated with the development of spheroid networks.

## Discussion

### Summary

We have described the process of spontaneous cluster formation of human induced pluripotent stem cell (iPSC)-derived neurons and astrocyte co-cultures on MEAs, and the corresponding development of network bursting activity for different cell densities and astrocyte ratios. Our data support an influence on the formation of cell clusters by astrocyte ratio, as well as the surface properties of the culture substrate as determined by prior use of the MEAs. Astrocyte ratio also influences the development of synchronized network activity, generally by suppressing network bursts while prolonging their duration. We observed an effect of cluster formation on network activity, where cluster formation accelerates the development of synchronized activity, shortens network bursts, and acts locally by producing more pronounced bursts on electrodes in proximity to large clusters. We observed that cell density is positively correlated with many aspects of network activity, most notably the correlation coefficients between units. It is worth pointing out that initial cell density does not reflect the local cell densities after clusters have formed.

### Role of astrocytes, cell density, and prior use of substrates in network activity and cluster formation

We showed that astrocyte density and prior MEA culture use are both major contributors to cluster formation. It is likely that multiple cycles of culture generate a more hydrophilic surface, facilitating adhesion. Treating surfaces with charged molecules is commonly used to reduce cell clustering (Maeda et al., 1995). The role of astrocytes in a formation of clusters suggests that the astrocytes exhibit or facilitate migratory behavior, a phenomenon that has been reported by others (Krencik et al., 2017). In mammals, the migratory behavior of astrocytes is poorly understood (Molofsky and Deneen, 2015), but astrocyte precursors are capable of migrating from their site of birth to most areas of the CNS where they terminally differentiate. Mature astrocytes can, in response to injury, become reactivated into a more migratory phenotype (Tezel et al., 2001), a behavior that underlies the formation of glial scars, which are known for preventing axonal regrowth in CNS injury and are a major target for potential therapy (Silver and Miller, 2004). Although reactivation of astrocytes is attributed to promoting an inflammatory and antiregenerating environment, several beneficial effects of astrocyte migration and reactivation have been uncovered (Renault-Mihara et al., 2008). It is possible that our cultured astrocytes are exhibiting reactivation, but the extent of which astrocytes inhibit or promote axonal regrowth or otherwise influence the formation of neuronal networks is unknown at this point.

### Astrocyte influence on network activity

Astrocytes are well known for their important homeostatic role, but also for their intimate involvement with neuronal synapses. The role of astrocytes in suppressing network bursts was initially surprising to us, as astrocytes are known to facilitate the formation of synapses in the developing cortex (Stogsdill et al., 2017). Astrocytes are essential for neuronal survival *in vivo* and are believed to be important for synapse formation, since astrocyte maturation coincides with a peak in synaptic formation in neonatal rats (Chung et al., 2015). Astrocytes are also important regulators of synaptic transmission and are closely associated with the synaptic clefts where they modulate multiple synaptic transmission systems (Tan et al., 2021). The importance of astrocytes in synaptic development has been demonstrated in *in vitro* settings similar to ours (Taga et al., 2019), and it has been shown that astrocytes are crucial for the generation of network bursts *in vitro* in the same cell line as the ones used in our experiment (Tukker et al., 2018). Because of the widely held view that astrocytes are key to neuronal function, experimenters often use astrocyte-conditioned medium to improve neuronal function (Fukushima et al., 2016).

It seems paradoxical that synchronous network events are more pronounced and more numerous at lower astrocyte densities. Astrocytes are essential for excitability through their selective uptake of glutamate from the synaptic cleft and secretion of glutamine to provide the required precursor for glutamate synthesis by neurons (Anderson and Swanson, 2000). This likely explains the dramatic increase in excitability when the same neuron types used here are co-cultured with astrocytes compared with neuronal monocultures (Tukker et al., 2018). It is possible then that at high astrocyte densities, reuptake of glutamate by more astrocytes leads to tighter regulation of extracellular glutamate concentration.

However, as network bursts are likely influenced by complex interactions of multiple systems (Masquelier and Deco, 2013), no simple parallel can be drawn between a single property of network bursts and synaptic strength or a single neuronal or astrocytic uptake mechanism. At this point, one can also not exclude the possibility that astrocytes are passively affecting the signal characteristics of the electrodes, either by insulation or by some other physical interaction. However, while individual spike detection may be affected by insulation, network bursts are very robust events, and discerning their presence and temporal dynamics seems unlikely to be hampered by passive properties of astrocytes. To examine this is not a straightforward task, however, as no perfect substitute for astrocytes that have identical passive properties is available at this point. One could imagine that merely the increased cell density reduces available energy sources for neurons. Future experiments that would elucidate the role of astrocytes would have to target specific systems, such as glutamate reuptake. Furthermore, one could attempt to grow the same proportions of astrocytes and neurons in close, but not direct, proximity. This could give us a clue as to whether the effect of astrocytes is by direct cell interactions, such as glutamate reuptake, or by indirect interactions through energy consumption or by secretion.

It is worth mentioning that, although neurons and astrocytes constitute a large portion of the cortical biomass, studies have shown enhanced network development by culturing primary rat neurons on surfaces coated with decellularized brain tissue (Lam et al., 2019), suggesting that some important aspects of brain extracellular matrix (ECM) may not be fully represented in traditional neuronal cultures, including ours.

### Cluster formation effects network bursts

Our observations all suggest a strong link between the morphology of the spheroid networks and the network activity of cultured neurons, but the mechanisms underlying this relationship are not directly discernible with our current data. It has been suggested that the relative abundance of nearby neurons could alter the dynamics of network behaviors (Frega et al., 2014). When clusters form, the relative distance that axons have to grow to find new postsynaptic contact candidates is shorter, thus accelerating the formation of strong synaptic circuits, which are presumed to underlie correlated activity (Chiappalone et al., 2006). On the other hand, the nerve-like bundles of parallel neuronal and astrocytic processes, which interconnect clusters in our cultures, are more sparsely populated by cell bodies, suggesting that action potentials would have to travel long distances in unmyelinated axons to reach postsynaptic targets in other clusters. This might be expected to reduce the correlation between distant electrodes, yet we observed the opposite. Interestingly, we see shorter network bursts in highly clustered networks, which is opposite to the effects observed in prior work for 3D cultures compared with an equivalent 2D culture (Frega et al., 2014). On the other hand, our model represents multiple local 3D structures interlinked by tract-like processes, whereas these prior 3D cultures effectively represent a single large cluster, with cells distributed uniformly throughout it. For cultures like ours with pronounced local clusters, the paths over which network bursts can travel is therefore more limited, making it impossible for them to reverberate throughout the culture for longer periods of time. It could be that less clustered cultures have, on average, greater distances and more directions for the network burst to travel, leaving time for more neurons to become ready for recruitment into the network burst again. It is also important to point out that a longer network burst does not necessarily translate directly into a stronger synaptic network. One could imagine that when a network is strongly connected, a network burst is quick to “exhaust” the network, while a more sparsely connected network does not immediately recruit all neurons into the burst. This idea is supported by our observation that burst durations generally decrease over time, although it is likely that more synapses are formed over time.

### Implications of network bursts

Many different forms of neuronal cultures are observed to develop synchronous bursting behavior, including insect neurons such as locust (Greenbaum et al., 2009) and mosquito (Gaburro et al., 2018), and mammalian neurons such as rat (Lam et al., 2019) and human (Heikkilä et al., 2009). Although bursts are almost ubiquitous in neuronal culture systems, human neurons have burst characteristics that set them apart from, for example, rat neurons (Hyvärinen et al., 2019). It seems to us that because network bursts are found in almost all types of neuronal cell cultures, the translational aspect should not be asserted without sufficient mechanistic understanding. Network bursts should perhaps be seen more as reporters of a large set of dynamic neuronal systems, including synapse formation (Brewer et al., 2009). This view is supported by the fact that network bursts can, depending on the cell type used, be influenced by modulation of a large set of receptor pathways, including glutamate signaling (Heikkilä et al., 2009), GABA signaling, cholinergic signaling (Dias et al., 2021), serotonergic modulation (Novellino et al., 2011), the actions of L-type and T-type calcium channels (Plumbly et al., 2019), and many other possible mechanisms.

Superficially, network bursts seen in our cultured neurons resemble disinhibited cortical network bursts (Menendez De La Prida et al., 2006) or induced epileptiform activity from cortical sections (Rutecki and Yang, 1998). However, further research needs to be conducted to elucidate the translational value of these cultures. The mechanistic similarities between network bursts and epileptiform activity of the brain can be investigated using antiepileptic drugs, substances known to cause epileptiform seizures, or even antiepileptic nutrient profiles such as those induced by ketogenic diets given to epileptic patients (D’Andrea Meira et al., 2019). Furthermore, it is possible to obtain iPSCs and subsequent neurons from individuals with genetic predisposition to epilepsy to investigate how these “epileptic” neurons differ in terms of network burst dynamics.

We mentioned previously that conventional neuronal organoids typically contain heterogeneous cell lineages in various stages of differentiation, which complicates the interpretation of treatments and culture conditions as so many confounding and highly dynamic systems influence network bursts. With the possible future experiments outlined above, this is perhaps especially relevant, as off-target effects and cascading or adaptive effects become more likely the more dynamic systems and cell types are involved. Using modern genetic approaches, such as siRNA or genetic or optogenetic constructs, these limitations can be overcome to some degree, but the simplicity of growing defined cell types that recapitulate a smaller set of dynamic systems and cell types is a more accessible approach.

Furthermore, network bursts share some similar features with the developing immature brain (Ben-Ari, 2001). In the developing neocortex, synchronized network activity influences many important aspects of cortical development including neurogenesis, differentiation, apoptosis, and development of functional neuronal networks (Kilb et al., 2011). Again, the relevance of these similarities is uncertain, but it is known that neuronal electrical activity influences a large range of neuronal physiological processes (Zhang and Poo, 2001), and, expectedly, these network bursts are likely having a strong influence on the behavior of the neurons. It is also important to note that many *in vivo* network activities display characteristic sinusoidal field potentials, which are occasionally observed, but not yet studied in this model.

### Potential pitfalls of spike sorting

We used sorted spike trains as the foundation of many of our analyses, but PCA-based spike sorting relies on neurons showing similar waveforms regardless of spike rates or other extrinsic or intrinsic factors. Using PCA-based sorting techniques is common practice, but there is no perfect technique for unsupervised clustering of spikes (Sukiban et al., 2019). This approach is perhaps even more questionable when neurons exhibit network bursts, as we expect an increase in overlapping spikes, which could alter waveform shape and thus their position in PCA space. To our knowledge, there is currently no established technique for overcoming this issue, and further research into spike sorting and assessing network dynamics in the presence of network bursts is needed.

### Limitations to spheroid networks

Above, we have described some of the benefits of using a defined differentiated population of neurons and astrocytes over using heterogeneous stem cell-derived organoids. While this limits the number of possible underlying mechanisms for any given observation, naturally, this also limits the range of potential mechanisms that we are able to study. Also, our use of predifferentiated neurons precludes functional analysis of specific developmental stages that could be observed by *in situ* differentiation of stem cell-derived cell populations.

A characteristic feature of the cortex is its layered structure, a feature that can be somewhat reproduced in unattached organoid cultures (Qian et al., 2020). We have not observed layer formation in our spheroid networks, likely as this formation relies on early morphogenic events, which establishes a radial morphology around the ventricular zones (Cadwell et al., 2019). The overall lack of comparable cytoarchitecture between spheroid networks and the cortex remains a challenge worth undertaking. Thankfully, there exists a wealth of techniques to manipulate neuronal morphology and connective characteristics (Millet and Gillette, 2012), and these techniques could provide effective means to improve neuronal *in vitro* models. In our opinion, too little attention is drawn toward the use of these techniques to model known neuronal circuits, such as the thalamocortical circuit, known for its importance in consciousness, epilepsy, and sleep (Steriade, 2005). Although capturing the full complexity of the human brain fits a certain purpose, it is known from computer simulations that relatively simple models can still exhibit, for example, gamma oscillations (Traub et al., 1997), a phenomenon that, to our knowledge, has only been reproduced *in vitro* on exceedingly rare occasions (Samarasinghe et al., 2021). This seems to suggest that the path toward a brain-on-a-chip lies in achieving appropriate cytoarchitecture and connective properties.

### Concluding remarks

Recent advances in 3D neuronal cultures often use artificial substrates or highly heterogeneous cell populations, whereas our model benefits from being sufficiently simple in cell population and being composed only of living material. The common approaches for 3D neuronal cultures today are organoids and soft ECM or hydrogel substrates. Although ECM-based and hydrogel-based approaches have been successful, they rely on either handcrafting hydrogels or obtaining ECM components from living tissue with the risk of batch variations. Generally, multiunit extracellular high-throughput recordings are challenging in such contexts as cells are not grown directly on electrodes, and solutions to this are promising but still in an early developmental phase (Soscia et al., 2020). While organoids are powerful in reproducing complex electrophysiological behaviors of the human brain (Yokoi et al., 2021), pluripotent cell-derived organoids often show large heterogeneity, posing a challenge for functional analysis in their sheer complexity (van den Hurk and Bardy, 2019).

To conclude, we argue that our model provides an ideal middle ground in complexity, between traditional 2D culture and more elaborate 3D organoids. Although it is a simple model with only three cell types, it nevertheless recapitulates many morphologic and physiological features observed in living brain tissue, while maintaining the convenience and physiological access to electrical activity traditionally associated with 2D cultures. The features that we describe, including long-range connections mediated by nerve-like structures and the patterns of bursting that resemble *ex vivo* models for epileptiform behavior, combined with the possibilities offered by iPSC technology suggest spheroid networks as a platform for further research, and for modeling the human cortex.

## Synthesis

Reviewing Editor: Benjamin Deneen, Baylor College of Medicine Decisions are customarily a result of the Reviewing Editor and the peer reviewers coming together and discussing their recommendations until a consensus is reached. When revisions are invited, a fact-based synthesis statement explaining their decision and outlining what is needed to prepare a revision will be listed below. The following reviewer(s) agreed to reveal their identity: Bruno DiStefano.

Overall, the reviewers felt this paper was of interest and had important merits. Nevertheless, there are a series of major and minor issues that the authors should address prior to publication of this paper. Below are the major issues that myself and the reviewers have discussed. Following these major points are the specific comments from each reviewer.

1) We all feel that use of the word "organic" is not appropriate, as these cell structures are not formal organoids and rather are derived from commercially available cell lines. Even though this is acknowledged in the intro, we feel that organoid should be deemphasized.

2) Much is made about GABA or glutamatergic neurons and activity. This is also somewhat misleading as the authors are not actually measuring IPSC, EPSC or recording from specific subtypes of neurons in these cultures. The recommendation is have them refrain from use of GABAergic or Glutamatergic and just stick with neuronal activity. Alternatively, authors could try to measure IPSCs and EPSCs or they can label neuronal subtypes (i.e. use Dlx5/6 promoter driven GFP for inhibitory neurons, etc) and record from labeled neurons. Finally, it would be good to know the constituency of the neuronal populations in these cultures. This can be achieved by staining for associated GABAergic or gutatmatergic makers and quantifying.

3) The authors need to run a quality control of the cell types from the commercially available vials to ensure they are correctly sourced.

4) It seems that low bursting TENDS to occur in the samples with Low Cell Density, irrespective of astrocyte number. Therefore the low bursting is probably due to low numbers of neurons in those cultures; this scenario should be included in the discussion. Also, with that said, the authors should do the suggested controls: culture with a control condition that has no astrocytes. This experiment has been done in retinal ganglion cells (Christopherson, et al. Science 2000) and basically there was no activity. This experiment would be a good fit here in this system.

Below are the reviewer comments that also need to be fully addressed:

Reviewer 1

This manuscript presents an approach to generating neuronal spheroids composed of glutamatergic neurons and astrocytes on micro-electrode arrays. Using this system, they record hundreds of neurons for up to 90 days. They discover that the number of astrocytes in the spheroids influences the electrophysiological network activity. Overall, the study is preliminary. The main conclusions of the manuscript could be strengthened by additional experiments, as outlined below:

- The authors state in the title, abstract, and throughout the paper that 3D brain organoids are used to perform the experiments presented. However, the authors are culturing commercially available astrocytes and neurons in monolayer as spheroid-like structures. The authors should avoid using the word brain organoid, which can be misleading, and use spheroids instead.

- Despite the authors mentioning "GABAergic networks" in the abstract, all the experiments presented in the result section are obtained using glutamatergic neurons. To strengthen the paper results, the authors should include data using GABAergic neurons.

- The finding that increasing the numbers of astrocytes has a detrimental effect on neuronal activity is counterintuitive. It would be interesting to test whether neurons cultured without astrocytes would still generate network activity. Moreover, the authors should test whether co-culturing neurons with other cell types (e.g., fibroblasts) would disrupt the neuronal networks.

- The paper would benefit from functional data. The authors should record neuronal activity using neurons derived from human iPSCs carrying mutations associated with neuronal disorders, such as individuals with a genetic predisposition to seizures in childhood.

- Statistical analyses are missing throughout the paper and should be included in the figures.

Reviewer 2

The authors of this manuscript have developed and tested a novel 3D culture system to study electrophysiology of neuronal activity ex vivo. This system aims to minimize complexity found in other 3D culture systems to facilitate the study of electrical activity. The culture consists of pre-differentiated neurons and astrocytes from iPSCs that are plated directly on micro-electrode arrays (MEAs). Four conditions were tested to measure the effects of astrocyte ratio and cell density on cluster formation and electrical activity of the network. This system was suitable for long-term culture of cells to measure electrical activity over 60+ days. While the authors provide data on many metrics of this system, several questions about the limitations and applications of the system remain unanswered.

Major suggestions:

1. The authors tested three cell types: glutamatergic neurons, GABAergic neurons (a smaller percentage than glutamatergic neurons), and astrocytes. The manuscript should mention the rationale and limitation of this approach compared to other organoid systems, including the heterogeneity of astrocytes observed in vivo.

2. The authors used commercial pre-differentiated cells in the study. Although the suppliers provided qPCR data classifying these cells as glutamatergic, confidence in the study could be increased with a secondary method to measure the purity of the cells. We recommend performing at least immunofluorescent staining to quantify the purity of cells for each batch if possible.

3. It is not clear how authors determined high versus low density. Do these reflect in vivo densities?

4. The authors used entropy to detect network activity features that cannot be detected by burst detection algorithms. However, it is not entirely clear what entropy evaluates.

5. In the text there is description about cultures suggesting that higher astrocyte ratios developed clusters faster, but there is minimal data (or data not shown) supporting it.

6. In all the graphs showing the neuronal activity during culture, particularly figure 4, there are data points missing (or not obvious due to the color scheme). For example, in figure 4C, there is no green bar by day 90. We recommend either change the dot color to match the color scheme or use lines to show the trend over time.

7. The manuscript concluded that astrocyte density is a major contributor to cluster formation. Was there a control condition in which astrocytes were not included in the culture? An additional control without astrocytes may also help address the surprising observation that astrocytes are suppressing network bursts in this system.

8. In the manuscript, the authors use pre-differentiated neurons, could this system be used to study neural development using stem cells and would this be more reflective of the brain?

9. Figure 1 shows co-localization of pre- and post-synaptic markers. What time point do these images represent? Does the amount of co-localization change over time, or does it vary between the four tested conditions?

In addition, there are a few minor issues in the manuscript:

- It is unclear if the word ‘organoid’ in this manuscript refers to each individual cluster, or all clusters on one MEA.

- Use of the word "organoid" is non-typical. How does the 3-D system in this manuscript compare to organoids grown in non-adherent conditions?

- Line 55: error in citation

- Section 2.5: Dilution factors for antibodies are not provided.

- Line 327-330: This sentence is long and unclear. Revising and breaking into two sentences may help ensure the intended message is not lost.

- The figures lack labels.
